# Dupilumab and House Dust Mite Immunotherapy in Patients with Atopic Dermatitis: A Preliminary Study

**DOI:** 10.3390/vaccines12091046

**Published:** 2024-09-13

**Authors:** Agnieszka Bogacz-Piaseczyńska, Andrzej Bożek, Magdalena Krupka-Olek, Aleksandra Kawczyk-Krupka, Jolanta Zalejska-Fiolka, Giorgio Walter Canonica

**Affiliations:** 1 Clinical Department of Internal Diseases, Dermatology and Allergology, Medical University of Silesia, 40-055 Katowice, Poland; agnieszka.anna.bogacz@gmail.com (A.B.-P.);; 2 Department of Internal Medicine, Angiology and Physical Medicine, Center for Laser Diagnostics and Therapy, Medical University of Silesia, Batorego 15 Street, 41-902 Bytom, Poland; akawczyk@gmail.com; 3 Department of Biochemistry, Faculty of Medical Science, Zabrze Medical University of Silesia, 40-055 Katowice, Poland; jzalejskafiolka@sum.edu.pl; 4 Head Personalized Medicine Asthma & Allergy Clinic-Humanitas Research Hospital, Humanitas University, 20089 Milano, Italy; giorgio_walter.canonica@hunimed.eu

**Keywords:** atopic dermatitis, immunotherapy, dupilumab, house dust mites

## Abstract

Background: Severe atopic dermatitis (AD) is a complex disease requiring systemic treatment. This study aimed to assess the effectiveness of combined therapy consisting of dupilumab and sublingual dust mite allergen immunotherapy (SLIT-HDM) in patients with severe AD and HDM allergies. Methods: Patients diagnosed with severe AD were included in this randomised, placebo-controlled, double-blind 12-month trial; they received SLIT for HDMs and/or dupilumab for 12 months and were compared with patients on cyclosporine. The primary outcomes for the treatment arms were changes in the Eczema Area and Severity Index (EASI), body surface area (%BSA), and Investigator Global Assessment (IsGA) over 12 months. The secondary outcomes were the proportion of patients who achieved IsGA success and reduced medication scores. Results: Significant improvements were observed in all analysed groups after 12 months of therapy based on the EASI, %BSA, and IsGA. However, the most substantial changes were observed in the groups treated with dupilumab or a combination of SLIT-HDM and dupilumab. Additionally, the proportion of patients who achieved an IsGA reduction was significantly greater in the group receiving combination therapy than in the other groups (9/14 [64% of the group receiving SLIT-HDM] vs. 11/14 [73% of the group receiving dupilumab] vs. 15/17 [88% of the group receiving dupilumab and SLIT-HDM] vs. 7/13 [53% of the group receiving cyclosporine]) (*p* < 0.05). Conclusions: In patients with severe AD and HDM allergies, combination treatment with dupilumab and allergen immunotherapy for HDMs may increase the therapeutic benefit over treatment with these methods separately.

## 1. Introduction

Atopic dermatitis (AD) is a chronic, recurrent inflammatory skin disease. It is a genetically determined skin disorder based on IgE reactions, damage to the epidermal barrier, inappropriate immune response, and abnormal microbial skin colonisation [1,2]. There are two immunological mechanisms of the disease: type I, the IgE-dependent allergic mechanism, and type IV, the allergic mechanism, which is dependent on allergen presentation to T lymphocytes facilitated by a specific IgE antibody in AD patients. There are often high concentrations of IgE in response to many food and inhalant allergens, including house dust mites, such as *Dermqatophagoides pteronyssinus* and *Dermatophagoides farina* [1]. Despite the relatively well-understood mechanism of the disease, treating patients with severe forms of AD, which, due to its complexity, may hinder the expected effects of therapy, is a challenge. The primary treatment for these patients is a biologic, which replaces current immunosuppressants [1,3,4]. In some cases, allergen immunotherapy is considered, limited mainly to desensitisation to house dust mites due to the allergic nature of AD [5,6]. However, despite several studies, more precise recommendations regarding the effectiveness of allergen immunotherapy for treating AD are currently needed [7,8,9,10]. In some allergic diseases, such as allergic rhinitis or allergic asthma, attempts have been made to combine biological treatment with AIT for a greater benefit [11,12,13]. Currently, no studies assess the effectiveness of combining allergy immunotherapy and biological treatment in patients with AD.

This study aimed to assess the effectiveness of combined therapy (SLIT-HDM + dupilumab) compared with individual therapies, SLIT-HDM alone, dupilumab alone, or cyclosporine, in patients with severe atopic dermatitis with an allergy to HDMs.

## 2. Methods

### 2.1. Study Design

This was a randomised, placebo-controlled, and double-blind interventional study, carried out in the Clinical Outpatient Allergy Department in Zabrze and six other outpatient cities between 2022 and 2023 (NCT06509243). All included patients received various therapy variants, including SLIT-HDM and/or dupilumab, for 12 months and were compared to patients on cyclosporine. Finally, sixty-five patients were randomised in comparable numbers to one of four arms of therapy with the use of a double-blind method. The randomisation procedure was performed with the use of computer-generated numbers. The number of patients included was based on a power calculation that took into account the expected effect size, the standard deviation of the results, and an ordinal variable for a comparative study of all study arms according to an appropriate formula.

### 2.2. Patients

Sixty-five patients were included in this study. Patients were eligible if they met the following criteria: between 18 and 45 years of age, had severe AD with an Eczema Area and Severity Index (EASI) > 20 points, a %BSA (body surface area) > 10 points, and an IsGA (Investigator Global Assessment) = 4 points, had a positive skin prick test (SPT) and a positive result for specific immunoglobulin E (sIgE) to *D. pteronyssinus* and *D. farinae* extracts and the major allergen Der p 1, had negative results for SPT and sIgE to remaining inhalant allergens, and had no symptoms of allergic asthma and/or allergic rhinitis. The severe AD diagnosis was based on the guidelines from the Polish Dermatological Society with more restrictive cut-off points for the EASI, %BSA, and IsGA scales, as presented in the inclusion criteria, to limit the study group to the most advanced forms of AD [14].

**Figure 1:**
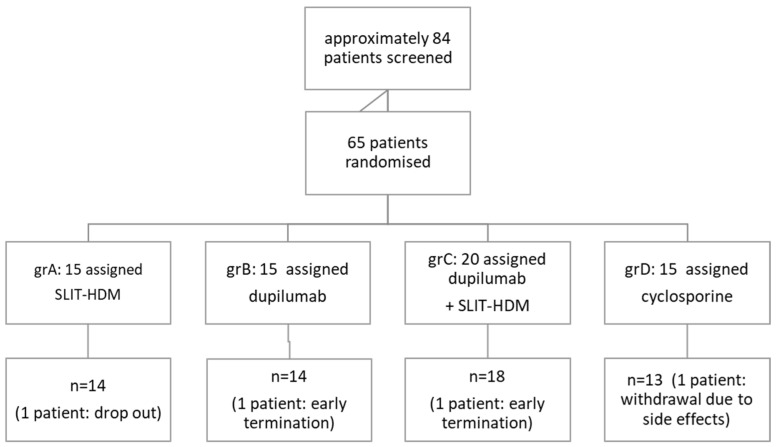
Flow diagram of the study patients. Legend: SLIT-HDM: sublingual immunotherapy for house dust mites.

Table 1 presents the patients’ characteristics, and Figure 1 shows a flow diagram of the patients.

The exclusion criteria included the following: other active dermatoses, systemic immunosuppressant treatment up to 7 months before the study, other chronic diseases, contraindications to sublingual immunotherapy or dupilumab, and lack of written consent. A dermatologist evaluated AD symptoms at each study visit.

### 2.3. Intervention

Patients were given access to four arms of therapy: 1. SLIT-HDM (Group A); 2. dupilumab (Group B); 3. SLIT-HDM + dupilumab (Group C); and 4. cyclosporine (Group D).

All treatments were blinded, and patients received, apart from the randomly assigned drug, depending on the arm, a blinded placebo of SLIT-HDM, a blinded placebo of dupilumab, and/or a blinded placebo of cyclosporine. An independent study coordinator performed the blinding and randomisation.

The SLIT-HDM therapy included ACARIZAX (ALk Abello, Horsholm, Denmark) with 12 SQ-HDMs of a standardised allergen extract of the house dust mites *D. pteronyssinus* and *D. farinae* (50/50%). The Acarizax tablets were removed from the blister unit immediately after the blister was opened and placed under the tongue, where they dissolved. Swallowing was avoided for approximately 2 min. Food and beverages were not ingested for 5 min after tablet intake. The daily dose was one tablet daily for 12 months.

Dupilumab treatment was administered according to established recommendations. The patient received a single initial dose of 600 mg of dupilumab and subsequent doses of 300 mg every 2 weeks.

Cyclosporine was administered at 3.5 mg/kg at the start of therapy, with the possibility of modifying the dose according to symptoms after a minimum of six months of therapy. Further treatment was administered for at least six months (12 months total). Treatment was discontinued if adverse effects occurred.

All patients used emollients. During therapy, patients with clinical signs of bacterially infected skin received treatment with topical mupirocin and/or a 7-day course of amoxicillin and/or prednisolone (0.5 mg/kg) for seven days for any occurrence of skin exacerbation, including a superinfection.

Depending on the case, oral antihistamines such as desloratadine and topical medications were added in all groups. If there was clinical worsening of AD (reported significant severity of itching, the appearance of erythroderma, and new skin lesions in a large area), the patient also received 10 mg of encortolon (glucocorticoid) every 7 days.

Symptomatic treatment was assessed using medication scores: 1 point for daily use of desloratadine, mometasone furoate cream, or mupirocin ointment; 7 points for every course of amoxicillin; and 10 points for a course of encortolon. The patients were required to record symptomatic drug use on the diary card.

### 2.4. Outcomes

The primary outcomes were the assessment of clinical improvement in AD patients during 12 months of interventional therapy, based on simultaneous evaluations of the following three scales, EASI, %BSA, and IsGA, in individual groups and between Groups A, B, C, and D from the beginning to the end of the 12th month.

The secondary outcomes were the proportion of patients who obtained IsGA success (meeting the condition: as a ≥2-grade improvement in IsGA from baseline), a reduction in the medication score, improvement in the Dermatology Life Quality Index (DLQI), changes in sIgG4 to *D. pteronyssinus* and *D. farinae* extracts, total IgE serum concentration, and the number of AD exacerbations (in terms of the need to use systemic steroids) during 12 months of observation. 

The Statistica version 8.12 (SoftPol, Cracow, Poland) was used to perform the analysis. Parametric or nonparametric tests were used depending on the data distribution. The ANOVA was used to compare the scale scores assessing the clinical condition of the skin. The Wilcoxon test was used to evaluate differences between the groups in some characteristic features. Differences were considered significant at *p* < 0.05.

## 3. Results

### 3.1. Clinical Improvement

**Figure 2:**
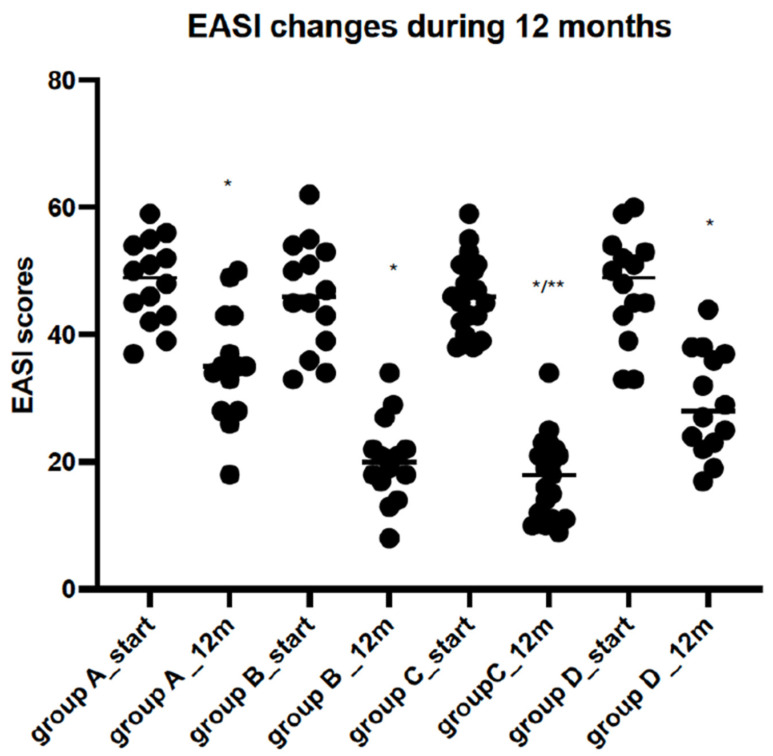
EASI score changes before and after 12 months of observation. Legend: Group A: patients receiving SLIT-HDM (sublingual immunotherapy to house dust mites), Group B: patients on dupilumab, Group C: patients on dupilumab and SLIT-HDM, Group D: patients on cyclosporine; *—a significant reduction in EASI score after 12 months of treatment in all study groups for *p* < 0.05; **—a more significant reduction in EASI score in Group C in comparison to Group B for *p* < 0.05 after 12 months of treatment (ANOVA test).

**Figure 3:**
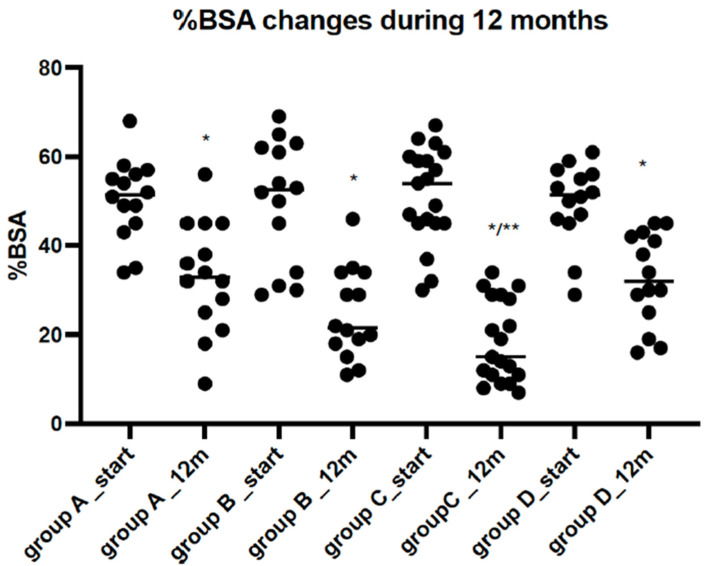
Body surface area (%BSA) before and after 12 months of observation in individual study Groups A, B, C and D. Legend: Group A: patients receiving SLIT-HDM (sublingual immunotherapy to house dust mites), Group B: patients on dupilumab, Group C: patients on dupilumab and receiving SLIT-HDM, Group D: patients on cyclosporine; *—a significant reduction in %BSA after 12 months of treatment in all study groups for *p* < 0.05; **—the greatest reduction was observed in Group C in comparison to Groups B, A, and D for *p* < 0.05 (ANOVA test).

**Figure 4:**
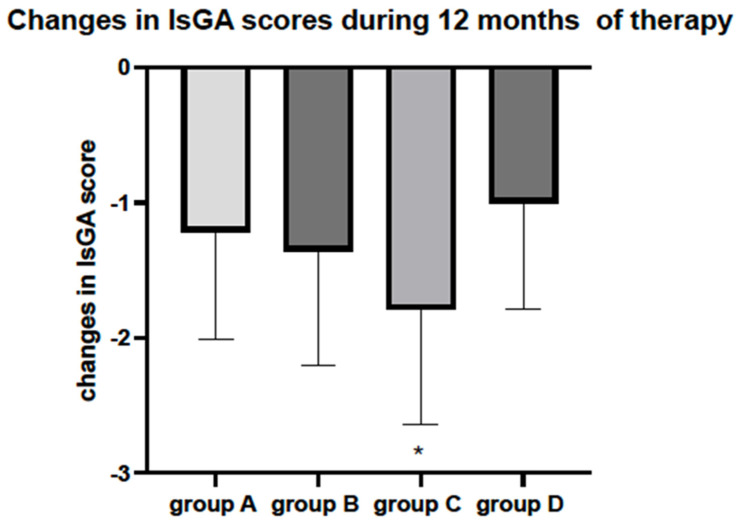
Changes in Investigator Global Assessment score before and after 12 months of observation. Legend: Group A: patients receiving SLIT-HDM (sublingual immunotherapy to house dust mites), Group B: patients on dupilumab, Group C: patients on dupilumab and receiving SLIT-HDM, Group D: patients on cyclosporine; a significant reduction in IsGA after 12 months of treatment in all study groups for *p* < 0.05. *—The greatest reduction was observed in Group C for *p* < 0.05 (ANOVA test).

**Figure 5:**
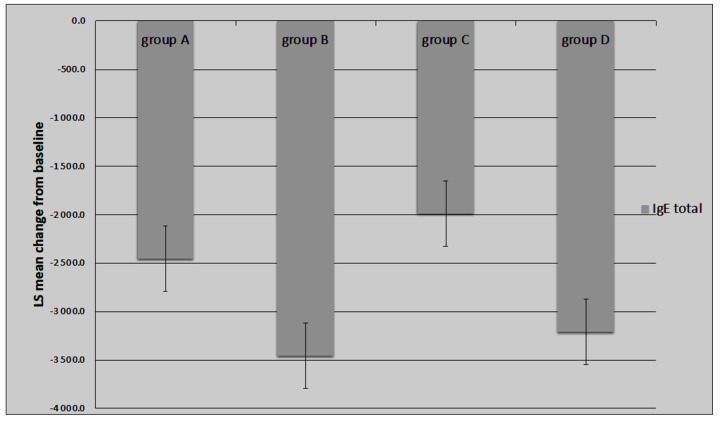
Decrease in total serum IgE at 12 months after the start of treatment. Legend: LS—Least Square Means; Group A: patients receiving SLIT-HDM (sublingual immunotherapy to house dust mites), Group B: patients on dupilumab, Group C: patients on dupilumab and receiving SLIT-HDM, Group D: patients on cyclosporine.

Significant improvements in the EASI, %BSA, and IsGA scores were observed in all analysed groups after 12 months of therapy. However, the most substantial changes in the tested scales were observed in the groups treated with a combination of SLIT-HDM and dupilumab (Group C) (Figure 2, Figure 3 and Figure 4). Additionally, the proportion of patients with an IsGA reduction was significantly greater in Group C than in the other groups: 9/14 (64% Group A) vs. 11/14 (73% Group B) vs. 15/17 (88% Group C) vs. 7/13 (53% Group D) for *p* < 0.05. Patients receiving dupilumab or combination therapy (Group C) reported a more significant improvement in quality of life on the DLQI scale than the remaining patients (Table 2). During 12 months of study, the number of AD exacerbations was significantly lower in Group C than in Groups A, B, and D: 2.1 vs. 1.7 vs. 0.8 vs. 2.2 per patient during 12 months of observation, respectively (*p* < 0.05). The medication score decreased significantly in all of the study groups; however, the most significant reductions were observed in Groups B and C, with a preference for the latter (Figure 5).

### 3.2. Immunological Parameters

**Figure 6:**
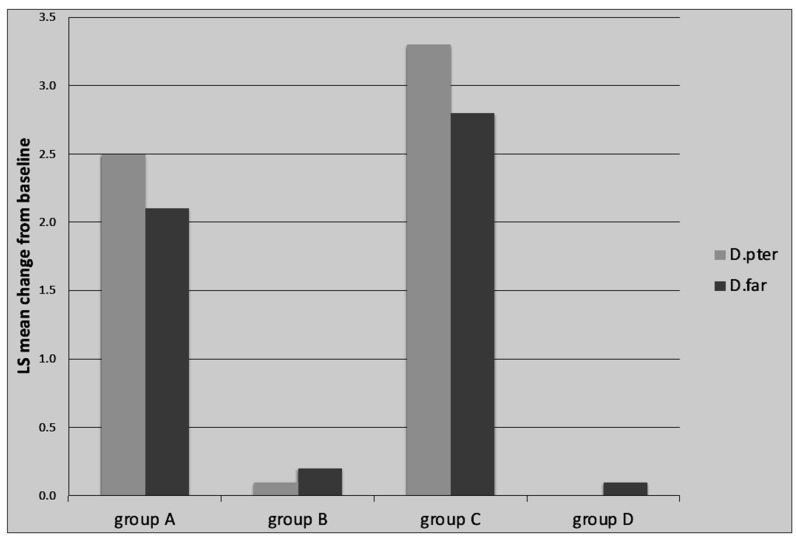
Increase in HDM-specific IgG4 levels 12 months after the start of treatment in the SLIT group. Legend: LS—Least Square Means; Group A: patients receiving SLIT-HDM (sublingual immunotherapy to house dust mites), Group B: patients on dupilumab, Group C: patients on dupilumab and receiving SLIT-HDM, Group D: patients on cyclosporine *D*. *pter*—mean change in IgG4 against *D*. *pteronyssinus* from baseline; *D*. *far*—mean change in IgG4 against *D*. *farinae* from baseline.

The mean total IgE serum concentration significantly decreased in all of the study groups (Figure 5). The levels of serum-specific IgG4 against *D. pteronyssinus* and *D. farinae* increased after a year of immunotherapy in study Groups A and C, which were treated with SLIT-HDM. The concentration of serum IgG4 in Groups B and D was consistently low compared with the other groups (Figure 6).

### 3.3. Safety

Three mild systemic reactions (grade 1 according to the Mueller classification: rash and/or episode of urticaria) requiring an antihistamine drug were observed during SLIT HDM. In addition, a mild local reaction was noted for 23 (0.1%) injections in eight patients in study Groups A and C. These reactions resolved spontaneously within 60 min following injections. In two patients (both in Group B), mild allergic conjunctivitis was likely a new symptom after dupilumab administration. These patients were treated with antihistamine ocular drops, with no recurrence.

## 4. Discussion

Treating severe AD has become easier in the era of biological therapy [14,15,16]. Dupilumab and other biologics significantly improve patients’ quality of life, often leading to completely reversing disease symptoms, especially compared with previous treatments [3,4]. In Europe, six systemic therapies for AD have been approved: the biologics dupilumab (anti-interleukin-4 receptor [IL-4R]), tralokinumab (anti-IL-13), and lebrikizumab (anti-IL-13) and the oral Janus kinase (JAK) inhibitors (JAKis) targeting JAK1/2 (baricitinib) and JAK1 (upadacitinib and abrocitinib) [4]. Dupilumab is a fully human monoclonal antibody that blocks the shared receptor component for IL-4 and IL-13, inhibiting the signalling of both IL-4 and IL-13, which are key and central drivers of type 2-mediated inflammation in multiple allergic diseases, especially in AD and asthma [17,18]. In patients with dust mite allergies, the effects of dupilumab can be complemented by creating tolerance through allergen immunotherapy to the allergens mentioned above, which leads to a shift in the balance from Th2 to Th1 immunological responses [19,20]. Therefore, dupilumab and allergen immunotherapy may synergise. Evidence of a similar enhancing effect was observed in combination with biological treatments, mainly omalizumab and immunotherapy, in patients with asthma or allergic rhinitis who, in addition to desensitisation to the allergen, were administered prolonged omalizumab [21,22,23]. In those studies, a more significant reduction in symptomatic therapy was achieved in patients with asthma treated with dupilumab and allergen immunotherapy than in those treated with these treatments alone [23]. However, most studies assessing the effectiveness of combined therapy with a biological drug and immunotherapy have focused on omalizumab. There are currently no published studies of AD except for isolated cases [24].

This study is the first to use a combination of biological therapy and SLIT-HDM in AD patients and evaluate its clinical effectiveness. The present original and novel results confirmed that patients treated with dupilumab and SLIT-HDM achieved better outcomes than patients in the other groups, as assessed by the EASI, %BSA, and IsGA scales, as well as by an improvement in quality-of-life (DLQI) scores and a reduction in typical symptomatic treatment and the number of disease exacerbations per year. Therapy with dupilumab alone was moderately worse for the EASI, %BSA, and IsGA assessments, followed by HDM-SLIT. This study revealed that cyclosporine treatment, although often effective, is less effective than treatment with dupilumab with or without SLIT-HDM. Notably, all of these therapies are associated with a strong safety profile and are consistent with data from the literature [3,23,25,26]. Unfortunately, no such data exist regarding the combination of dupilumab with SLIT-HDM. Some studies have assessed the effectiveness of treating severe AD in patients allergic to HDMs via allergen immunotherapy [5,27,28,29,30].

In the literature, some studies focused on patients with AD and AIT-HDM, and most of them confirm the effectiveness of such treatment, especially in mild and moderate forms of AD [31].

In a retrospective study, patients with moderate-to-severe AD received SCIT HDM and pharmacotherapy for three years (SCIT group) or only pharmacotherapy (non-SCIT group). After three years of treatment, the SCORAD and pruritus VAS scores significantly decreased in the SCIT-HDM group [27]. Similar studies have been conducted in children with AD and HDM allergies. The SLIT group received SLIT with *D. farinae* and pharmacotherapy or pharmacotherapy alone. After three years, the SCORAD scores in the SLIT group were significantly lower in comparison to the control (*p* < 0.05). The authors confirmed that this immunotherapy was safe and effective in paediatric patients with AD. The effectiveness was maintained even after treatment cessation [32].

However, most of the studies on the efficacy of AIT-HDM concern patients with AD and comorbidities: allergic asthma and allergic rhinitis. In many of them, not only was a reduction in respiratory allergy symptoms observed but also a clinical improvement of the skin and a reduction in itching. These observations concerned both the injectable and sublingual methods of immunotherapy to HDMs [33,34]. However, the subgroups of patients with concomitant AD were not dominant in these studies. There is a lack of similar studies in patients with AD and confirmed allergies to allergens other than HDMs. Also, evaluating the efficacy of AIT in patients with AD and polyvalent allergies requires further studies.

To summarise, opinions on the effectiveness of immunotherapy against AD mites vary, but the prevailing concerns are the benefits of this treatment. However, there are no studies on large groups of patients who only exhibit severe AD and monosensitisation. Further research is needed to evaluate the role of allergen immunotherapy in severe AD treatment.

This study has significant limitations. First, the sample size was small because of the restrictive inclusion criteria, including monovalent allergies to house dust mites. However, this approach made it possible to obtain a homogeneous group of study patients, which allowed for the effectiveness of the therapy to be rigorously tested. The cases involved a very advanced stage of the disease with simultaneous monovalent allergies to mites. Second, this study did not use a placebo for ethical reasons; the control group (Group D) consisted of patients who used a recognised, although older, treatment method such as cyclosporin. This comparison allows for a reliable assessment of new treatment methods: (1) the recognised treatment, dupilumab, which performs better, in line with expectations and data from the literature, and (2) the novel combination therapy. Notably, most patients showed significant improvement with all treatment methods, emphasising their reliability and the appropriateness of this research project. Another important factor that may have had an impact on the outcome is the use of topical steroids and/or oral steroid therapy in severe AD and their long-term impact on the skin condition of patients. Although the use of these medications was included in the total medication score, these results may have been underestimated. Another problem was the short observation time; however, this is an ongoing study, and long-term results will be available. The use of combination therapy should be dedicated only to a specific group of AD patients. Still, this study only allows for the definition of such a target group, owing to the small number of participants. In addition to the current evidence on the effects of combination therapy, further studies that include patients who would benefit most from combination therapy are necessary.

## 5. Conclusions

In patients with severe AD and house dust mite allergies, combination therapy with dupilumab and allergen immunotherapy for HDMs may increase the benefit of treatment compared with using these treatments separately. However, further observations are needed.

## Figures and Tables

**Table 1:** Patient characteristics at the start of the study.

Characteristics	Group A (*n* = 15)	Group B (*n* = 15)	Group C (*n* = 20)	Group D (*n* = 15)
Age (mean)	20.9 ± 2.4	23.1 ± 5.1	19.2 ± 7.4	20.9 ± 6.6
Women (%)	38	41	42	35
EASI				
Mean ± SD	47 ± 5.2	45 ± 6.2	50 ± 8.8	51 ± 9.2
Average	38–58	40–59	38–54	34–52
%BSA				
Mean ± SD	58 ± 12	62 ± 10	60 ± 14	55 ± 9
Average	40–81	39–82	33–81	41–82
Duration of AD (years)	12.1 ± 5.2	14.3 ± 2.9	14.9 ± 6.2	11.2 ± 8.9
Number of patients with nonallergic asthma	0	0	0	1
Number of patients with rhinitis	0	1	0	0
Number of smokers	0 ^†^	3	4	3
Total IgE (kU/L) SD	9231 ± 2544	11,460 ± 5322	8500 ± 4791	10,203 ± 3421
sIgE to D. pter (kU/L) SD	12.4 ± 2.4	8.9 ± 4.2	19.3 ± 5.3 ^†^	10.1 ± 4.1
sIgE to D. far (kU/L) SD	8.1 ± 4.9	10.2 ± 3.3	9.4 ± 1.7	3.9 ± 1.4 ^†^
Treatment before study ^‡^, *n* (%)				
Antihistamines	15 (100)	15 (100)	20 (100)	15 (100)
Topical glucocorticosteroids	15 (100)	13 (87)	19 (95)	15 (100)
Calcineurin inhibitors topically	12 (80)	12 (80)	17 (82)	11 (73)
Systemic glucocorticosteroids	6 (40)	4 (27) ^†^	8 (40)	5 (33)
Cyclosporine	2 (62)	0 (62)	1 (62)	0 (62)
Methotrexate	1 (7)	1 (7)	1 (5)	2 (14)
Dupilimab	0	0	0	0
PUVA	9 (60)	10 (67)	14 (70)	11 (73)

Abbreviations: AD: atopic dermatitis; BSA: body surface area; Der f-D: farinae; Der p-D: pteronyssinus; EASI: the Eczema Area and Severity Index; IsGA: Investigator Global Assessment was 4 points in every patient; SD: standard deviation; sIgE: allergen-specific IgE. Notes: ^†^ Significant difference between patients in this group and those in all other groups (Wilcoxon or ANOVA tests). ^‡^ Therapy in the last 12 months.

**Table 2:** The Dermatology Life Quality Index (DLQI) and total medication score (TMS) results.

Type	Group A	Group B	Group C	Group D
DLQI (SD) before	12.1 (2.3)	10.9 (2.7)	12.6 (1.8)	11.4 (1.5)
DLQI (SD) 12 m	8.7 (1.4) ^†^	5.1 (2.1) ^†,^***	4.3 (0.77) ^†,^***	6.9 (5.1) ^†^
TMS (SD) before	2.23 (0.23)	2.42 (0.55)	2.18 (0.42)	2.58 (0.27)
TMS (SD) 12 m	1.45 (0.16) ^†^	1.13 (0.21) ^†,^***	0.81 (0.31) ^‡,^***	1.31 (0.41) ^†^

Abbreviations: DLQI: Dermatology Life Quality Index score; SD: standard deviation; TMS: total medication score; 12 m: after 12 months; ^†^ *p* < 0.05 compared with baseline; ^‡^ the largest reduction among the studied groups; *** the more significant reduction in Group C in comparison to Group B after 12 months of observation for *p* < 0.05 (ANOVA test).

## Data Availability

The data presented in this study are available upon request from the corresponding author. Due to ethical restrictions, they are not publicly available.
